# *In vitro* Analysis of Neurospheres Derived from Glioblastoma Primary Culture: A Novel Methodology Paradigm

**DOI:** 10.3389/fneur.2013.00214

**Published:** 2014-01-07

**Authors:** Lorena Favaro Pavon, Luciana C. Marti, Tatiana Tais Sibov, Suzana M. F. Malheiros, Reynaldo Andre Brandt, Sergio Cavalheiro, Lionel F. Gamarra

**Affiliations:** ^1^Departamento de Neurologia e Neurocirurgia, Universiade Federal de São Paulo (UNIFESP), São Paulo, Brazil; ^2^Hospital Israelita Albert Einstein (HIAE), Instituto do Cérebro (InCe), São Paulo, Brazil; ^3^Hospital Israelita Albert Einstein (HIAE), Centro de Pesquisa Experimental (CPE), São Paulo, Brazil; ^4^Programa de Imunopatologia e Alergia da Faculdade de Medicina da USP (FMUSP), São Paulo, Brazil; ^5^Hospital Israelita Albert Einstein (HIAE), Centro de Neuro-Oncologia, São Paulo, Brazil; ^6^Hospital Israelita Albert Einstein (HIAE), Neurocirurgia, São Paulo, Brazil; ^7^Faculdade de Ciências Médicas da Santa Casa de São Paulo, São Paulo, Brazil

**Keywords:** glioblastoma, primary culture, neurosphere/subspheres, adherent stem cell, CD133, methods

## Abstract

Glioblastomas are the most lethal primary brain tumor that frequently relapse or progress as focal masses after radiation, suggesting that a fraction of tumor cells are responsible for the tumor regrowth. The identification of a brain tumor cell subpopulation with potent tumorigenic activity supports the cancer stem cell hypothesis in solid tumors. The goal of this study is to determine a methodology for the establishment of primary human glioblastoma cell lines. Our aim is achieved by taking the following approaches: (i) the establishment of primary glioblastoma cell culture; (ii) isolation of neurospheres derived from glioblastoma primary cultures; (iii) selection of CD133 cells from neurospheres, (iv) formation of subspheres in the CD133-positive population, (v) study of the expression level of GFAP, CD133, Nestin, Nanog, CD34, Sox2, CD44, and CD90 markers on tumor subspheres. Hence, we described a successful method for isolation of CD133-positive cell population and establishment of glioblastoma neurospheres from this primary culture, which are more robust than the ones derived straight from the tumor. Pointed out that the neurospheres derived from glioblastoma primary culture showed 29% more cells expressing CD133 then the ones straight tumor-derived, denoting a higher concentration of CD133-positive cells in the neurospheres derived from glioblastoma primary culture. These CD133-positive fractions were able to further generate subspheres. The subspheres derived from glioblastoma primary culture presented a well-defined morphology while the ones derived from the fresh tumor were sparce and less robust. And the negative fraction of CD133 cells was unable to generate subspheres. The tumor subspheres expressed GFAP, CD133, Nestin, Nanog, CD44, and CD90. Also, the present study describes an optimization of neurospheres/subspheres isolation from glioblastoma primary culture by selection of CD133-positive adherent stem cell.

## Introduction

Nervous system (NS) tumors are classified into seven categories that include primary tumors, according to World Health Organization (WHO) (neuroepithelial tissues, meninges, cranial and paraspinal nerves, germ cell and sellar region tumors, lymphoma, and hematopoietic neoplasms), besides secondary or metastatic tumors.

Central Brain Tumor Registry of the United States of America (CBTRUS 2008/2011) data shows that 64,530 new cases of primary NS tumors were diagnosed in 2010, which represents 1.44% of all malign neoplasms diagnosed in the USA. In spite of the low incidence, the primary NS tumors are highly lethal and accounted for 13,140 deaths in USA during 2010.

Usually, central NS tumors are classified as gliomas and non-gliomas. There are several different types of gliomas, which are determined by the glial cell that give rise to the tumor. Astrocytoma, oligodendroglioma, glioblastoma, oligoastrocytoma, and ependymoma are examples of gliomas. Gliomas are 31% of the primary tumors and 80% of the NS malignant tumors. Glioblastoma, which is a subset of glioma (GBM, WHO astrocytoma grade IV) is the most frequent and malignant brain tumor. Although many advances have been made in these tumors diagnosis, glioblastoma generally has a very poor prognosis (1, 2).

Glioblastoma treatment is difficult due to some tumor features such as: (i) extensive tumor infiltration in the surrounding healthy brain tissue; (ii) tumor mass formed by distinct cell types with multiple cell markers; (iii) resistance to chemo and radiotherapy (3). These characteristics have driven investigators to hypothesize that glioblastoma is a heterogeneous combination of cells and cancer stem cells. Cancer stem cells are defined as a small fraction of tumor-initiating cells which have a high tumorigenic potential and proliferation rate (3, 4).

The CD133 has been used as a marker to identify regular neural stem cells (5), and also tumor neural stem cells. However there are three items used to confirm if the CD133 isolated cells are tumor stem cells instead regular ones: (1) generation of clonogenic cells (neurospheres); (2) proliferative and self-renew ability; (3) capacity of behaving as tumor initiators when implanted in immunodeficient mice.

Quantitative analysis of CD133-positive cells has generally found them to be present at low levels in human gliomas, glioma neurosphere, and established glioma cell lines (6), consistent with the assumption that tumor stem cells are a rare cell population in solid tumors. However, some studies reported an exceptionally high CD133 (20–60%) fraction in some human glioblastoma and/or glioma cell lines (7), and other authors also have reported that glioblastoma contain more than 25% of CD133-positive cells (8).

Facchino and coworkers (9) described that tumor brain purified CD133 cells are able to generate neurospheres in culture and also differentiate into glial cells and neurons in presence of inductors. The glioblastoma CD133-positive cells can generate highly invasive tumors *in vivo* (10–12), that are chemo resistant, radio resistant and therefore responsible for tumor progression and relapse after conventional therapy (2, 13).

The true role of CD133 in the initiation and progression of brain tumors is still an unclear process. Even though there are several articles that refers CD133 as the main tumor stem cell marker, still exists some differences among published articles regarding standardization methods. For example, there are several studies that have isolated stem cells from primary glioblastoma, using CD133 magnetic microbeads and obtained neurospheres by the use of supplements such as N2, B27, EGF, FGF with variable results (3, 12, 14). On the other hand, there is a group that isolated regular neural stem cell from a health volunteer using an adherent cells protocol with very good results (15). Thus, is highly desirable to establish standard methods for isolation, culture, and cellular characterization of neurospheres and subspheres aiming future studies of these cells tumorigenicity.

In the present study, we developed a modified procedure for neurospheres and subspheres isolation from human glioblastoma primary culture.

## Materials and Methods

In this study, we analyzed five samples of glioblastoma obtained from patients submitted to brain tumor surgery removal procedures in the Neuro-Oncology Center of the Hospital Israelita Albert Einstein (HIAE). All patients signed an informed consent for the study (Ethical Committee of the HIAE approved by number 687).

The brain tumors diagnoses were performed by the Integrated Neuro-oncology program team from HIAE based on MRI data and histopathology analysis (16). The histopathology data classified all tumors as GFAP positive, categorizing them as grade IV.

### Establishment of primary cell culture of human glioblastoma samples

Fresh glioblastoma samples were washed and minced in PBS (1×) followed by enzymatic dissociation with collagenase-I 0.3% (Sigma-Aldrich). The isolated cells were resuspended in Dulbecco’s Modified Eagle’s Medium-Low Glucose (DMEM-LG, GIBCO Invitrogen) supplemented with l-Glutamine 200 mM, Antibiotic–Antimycotic (10,000 U/mL sodium penicillin, 10,000 μg/mL streptomycin sulfate, and 25 μg/mL amphotericin B – GIBCO/Invitrogen Corporation), and 10% Fetal Bovine Serum (GIBCO/Invitrogen Corporation). Next, the cells were seeded in 25 cm^2^ cultures flasks and maintained at 37°C, 5% CO_2_. The culture medium was changed every other day.

### Glioblastoma neurospheres culture derived from tumor primary culture

The cells obtained in the primary culture described above were resuspended in *tumor brain stem cells medium* which is Dulbecco’s Modified Eagle Medium/F12 (Gibco), supplemented with N2 (Gibco), EGF (20 ng/mL, Invitrogen), bFGF (20 ng/mL, Gibco), leukemia inhibitory factor 10 ng/μL (LIF; Chemicon), and B27 (1:50; Life Technologies). Viable cells were seeded in 24-well plates at 2 × 10^4^ density. The cells were maintained in a humidified incubator (Thermo Fisher Scientific Inc. 3110, Waltham, MA, USA) with 5% CO_2_ at 37°C and culture medium were changed every 3 days.

### Glioblastoma neurospheres culture derived from fresh tumor

Fresh glioblastoma samples were washed and minced in PBS (1×) followed by enzymatic dissociation using collagenase-I 0.3% (Sigma-Aldrich). Cells were resuspended in tumor brain stem cells medium. Viable cells were seeded in 48-well plates at 1 × 10^2^ density. The cells were maintained in a humidified incubator (Thermo Fisher Scientific Inc. 3110, Waltham, MA, USA) with 5% CO_2_ at 37°C and culture medium were changed every 3 days (17).

### Immunocytochemical staining of CD133 cells glioblastoma

Glioblastoma tumor samples were washed with 1% PBS and tumor cells were disaggregated in 1% PBS solution containing 0.3% collagenase-I. Cells derived from the tumor were then resuspended in supplemented Dulbecco’s Modified Eagle’s Medium (DMEM-Low Glucose) and plated at a density of 3 × 10^6^ live cells/60 mm plate. Next, cells were fixed with 4% paraformaldehyde and stained with antibody against human CD133/1 (human monoclonal IgG1; 1:1000 dilution; Miltenyi Biotec). After washing the cells, they were incubated with KIT Advanced ™ HRP Dako (K4067) Advanced ™ HRP Enzyme and followed by the application of the substrate-chromogen solution (DAB^+^).

### Purification of brain tumor stem cells using CD133 microbeads

The neurospheres colonies were dissociated using StemPro Accutase (Invitrogen) and maintained at room temperature for 10 min. The cells were labeled with CD133 magnetic microbeads (MACS – Miltenyi Biotech) and selected by affinity column according manufactory instructions (Miltonic Biotech). In order to verify the cells populations separation efficiency, the CD133^+^ fraction was stained with CD133/2PE and evaluated by flow cytometry FACSARIA (BD Biosciences, San Jose, CA, USA) and FACSDIVA software (BD Biosciences, San Jose, CA, USA) (18).

### Transmission electron microscopy of glioblastoma CD133^+^ cells

After purification process an aliquot of CD133-positive cells fraction was also analyzed by Transmission electron microscopy (TEM) (18). The cells were fixed in 1% glutaraldehyde and 0.2 M cacodylate buffer for 2 h at 4°C. Cells were washed in cacodylate buffer, 2 times for 15 min each. Post-fixation was performed in 1% osmium tetroxide for 1 h at 4°C, followed by another two washes in the same buffer. For contrast, the pellet was immersed in a solution of uranyl acetate in acetone for 30 min. After dehydration, the material was embedded in Epon resin diluted in acetone (1:1) and incubated at 4°C with agitation for 24 h. The pellet was then transferred to pure Epon resin and incubated at 60°C for 72 h, until completely polymerized. Semi and ultrathin sections were obtained through Porter Blum ultramicrotome. The semithin sections were stained with azur II (1%) and methylene blue (1%). The ultrathin sections were placed on copper grids and stained with uranyl acetate and lead citrate. The grids were studied and photographed under a TEM (PHILIPS CM100).

### Preparation of tumor subspheres

CD133^+^ selected and CD133^−^ depleted cells populations, derived from column affinity purification, were resuspended in tumor brain stem cells medium and seeded in 24-well plates at 1 × 10^3^ density. Formation of subspheres was only observed in CD133-positive cells and documented by phase-contrast microscopy (Olympus IX51).

### Immunophenotyping of tumor subspheres by flow cytometry

We analyzed cell-surface expression of specific markers in subspheres cells. We used monoclonal antibodies commercially available and followed the manufacturer’s instructions. Briefly, subspheres were harvested by treatment with StemPro Accutase Cell Dissociation Reagent (GIBCO-Invitrogen, Carlsbad, CA, USA), washed with PBS (pH = 7.4). Next the cells were stained with the selected monoclonal antibodies, and incubated in the dark for 30 min at room temperature. For intracellular staining the cells were first fixed (Facs lysing solution, BD) and permeabilized (permeabilization solution 2, BD Biosciences, San Jose, CA, USA). The following human antibodies were used: GFAP PE (clone: 51-10C9; BD Pharmingen, San Diego, CA, USA), Nestin APC (clone: AD2; BD Pharmingen, San Diego, CA, USA), CD133-PE (clone: 133/2; Miltenyi biotec, Bergisch Gladbach, Germany), *Sox*2 PerCP-Cy5.5 (clone: 245610; R&D System), CD34 PE (clone: 581; BD Pharmingen, San Diego, CA, USA), Nanog PE (clone: 781; BD Pharmingen, San Diego, CA, USA), CD44 PerCP-Cy5.5 (clone: IM7; BD Pharmingen, San Diego, CA, USA), CD90 APC (clone: 5E10; BD Pharmingen, San Diego, CA, USA), and the related isotype controls. The data acquisition was done by FACSARIA flow cytometer (BD Biosciences, San Jose, CA, USA) and analyzed by FACSDIVA software (BD Biosciences, San Jose, CA, USA) or Flow Jo Software (TreeStar, Ashland, OR, USA).

### Immunofluorescence assay

To the immunofluorescence assays, the subspheres were fixed with formaldehyde 4%, permeabilized with 0.1% Triton X-100. The samples were incubated with the following fluorescently labeled antibodies: GFAP-FITC (1:100; Santa Cruz), CD133-PE (1:100; Miltenyi biotec, Bergisch Gladbach, Germany), Nestin-PE (1:200; BD Bioscience), and Nanog FITC (1:400; R&D System). The cells were also stained with 4′, 6′-diamidino-2 phenylindole (DAPI; Sigma). These cells were analyzed by fluorescence microscopy (Olympus IX51).

## Results

### Establishment of primary cell culture from human glioblastoma samples shows the presence of CD133-positive cells

**Figure 1:**
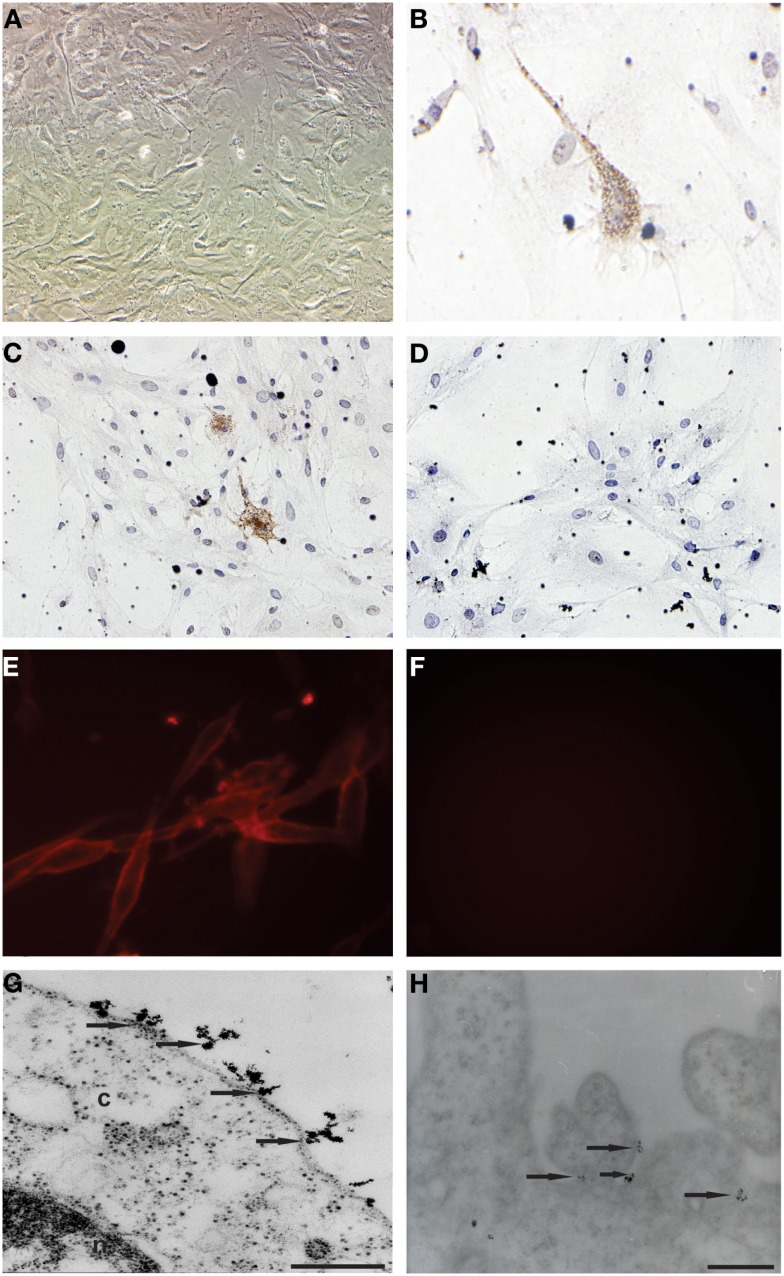
**Establishment of primary cell culture of human glioblastoma samples: immunocytochemical staining and transmission electron microscopy (TEM) of CD133^+^ cells glioblastoma**. **(A)** Analysis of primary culture of glioblastoma. **(B,C)** Immunolocalization of CD133^+^ cells by immunocytochemical staining. **(E)** Immunolocalization of CD133^+^ cells immunofluorescence assays **(G,H)** Immunolocalization of CD133^+^ cells by transmission electron microscopy and that arrows indicate electron-dense nanoparticles in the glioblastoma cell surface recognizing the CD133 epitope. **(D,F)** Glioblastoma cells from control group of immunocytochemical and immunofluorescence techniques, respectively. Representative figure of five samples of glioblastoma. **(A)** Magnification 400×. **(B–F)** Magnification 600×. Scale bars: **(G,H)** 0.25 μm.

Primary cell cultures were obtained from glioblastoma samples. These cells were homogenous, presented fusiform format, and were arranged in multidirectional bundles in culture (Figure 1A). Immunocytochemical (Figures 1B,C) and immunofluorescence (Figure 1E) assays revealed the CD133 expression in glioblastoma primary cultures. The Figures 1D and 1F represented glioblastoma cells from control group of immunocytochemical and immunofluorescence techniques, respectively. Ultrastructural analysis using electron microscopy highlighted the presence of electron-dense granules in the glioblastoma cell surface. These granules are compatible with the presence of anti-human CD133 microbeads attached to the cell membrane (Figures 1G,H).

### Glioblastoma primary cultures generate neurospheres with superior phenotype than those isolated from fresh tumor

**Figure 2:**
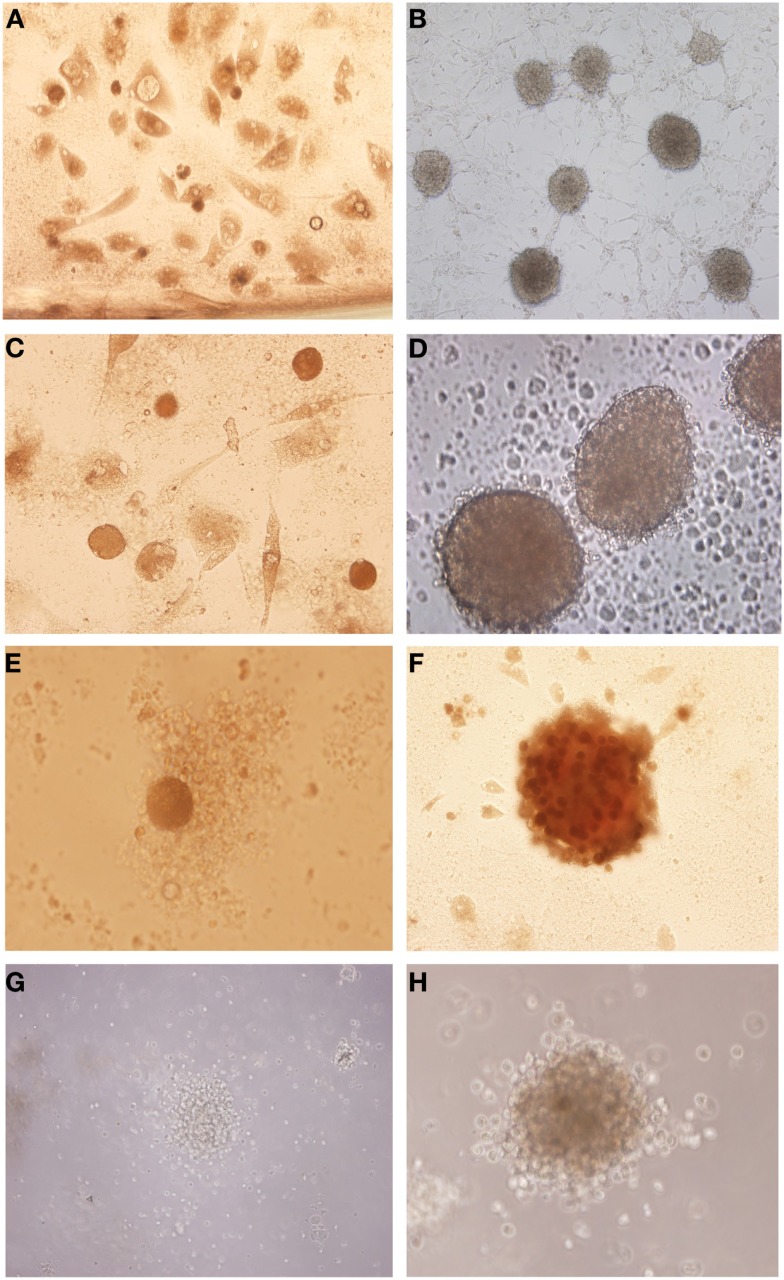
**Isolation of tumor neurospheres derived from glioblastoma primary culture and derived straight from the tumor**. **(A,C,E,G)** Glioblastoma neurospheres culture derived from fresh tumor. **(B,D,F,H)** Glioblastoma neurospheres culture derived from tumor primary culture. Representative figure of five samples of glioblastoma. **(A,B)** Magnification: 100×, **(C)** magnification: 200×, **(D–H)** magnification: 600×.

After the glioblastoma digestion the cells were resuspended in tumor brain stem cells medium and in culture generated neurospheres. However the cells derived from glioblastoma primary culture generated neurospheres with more developed and robust morphology (Figures 2B,D,F,H) compared with those isolated straight from the fresh tumor (Figures 2A,C,E,G).

### CD133 microbeads purified neurospheres are able to generated tumor subspheres

**Figure 3:**
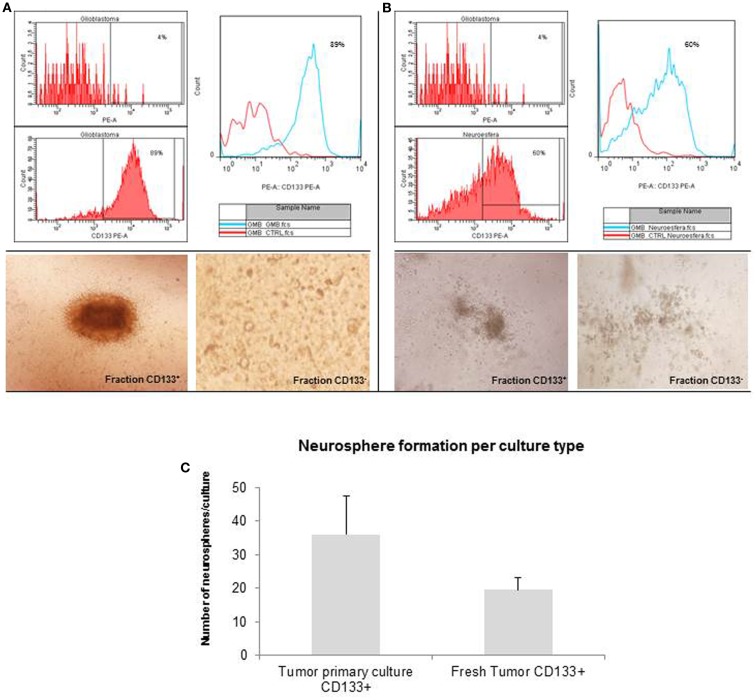
**Purification of brain tumor stem cells using CD133 microbeads followed preparation of tumor subspheres**. Immunophenotypic characterization by flow cytometry tests, evaluating the efficiency of magnetic separation of cell fractions positive for the antigenic marker CD133 in neurosphere derived from glioblastoma primary culture – 89.0% **(A)** and derived from fresh tumor – 60.0% **(B)**. CD133^+^ cells in neurospheres derived from glioblastoma primary culture were able to further generate subspheres. Culture of glioblastoma subspheres, compared with the absence of subspheres obtained from CD133^−^ fractions **(C)**. Graphic representation of neurospheres formation per culture type, such as tumor primary culture CD133^+^ and fresh tumor CD133^+^. Representative figure of five samples of glioblastoma. Magnification: 600×.

The glioblastoma neurospheres were treated with *StemPro Accutase* and selected by CD133^+^ affinity column. The neurospheres were evaluated for CD133 purity, and the glioblastoma primary culture derived neurospheres were 89% purity (Figure 3A) while the tumor fresh isolated neurospheres presented only 60% purity (Figure 3B). These results show a higher concentration of CD133-positive cells in neurospheres derived from glioblastoma primary culture. These CD133-positive fractions were able to further generate subspheres. The subspheres derived from glioblastoma primary culture presented a well-defined morphology while the ones derived from the fresh tumor were diffuse and less robust. Also, CD133-positive cells derived from glioblastoma primary culture were able to generate 75% more subspheres than the ones derived from the fresh tumor (Figure 3C). The negative fraction of CD133 cells were unable to generate subspheres (Figures 3A,B).

### Immunophenotyping of tumor subspheres by flow cytometry and fluorescence microscopy

**Figure 4:**
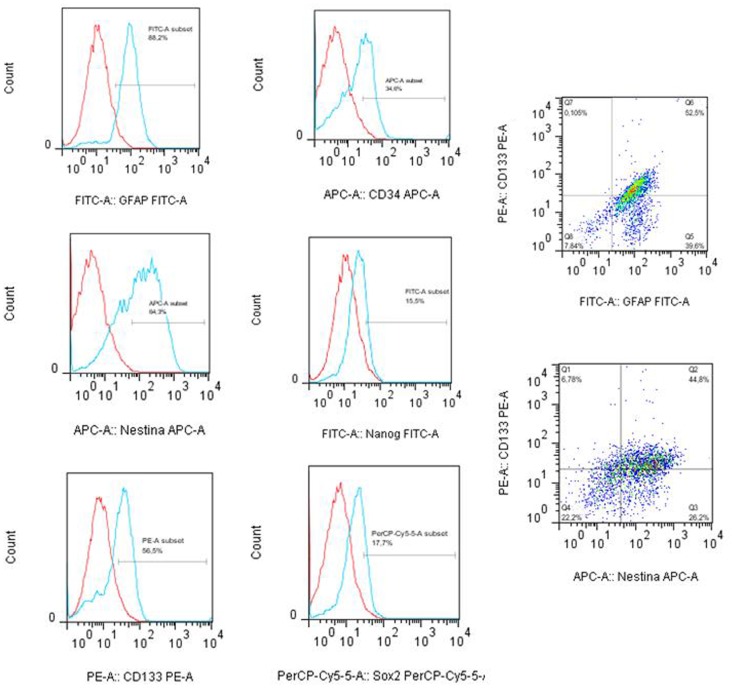
**Immunophenotyping of tumor subpheres by flow cytometry**. Immunophenotypic characterization by flow cytometry assays showing the pattern of expression of markers GFAP (88.2%), CD133 (56.5%), Nestin (64.2%), Sox2 (17.7%), CD34 (34.0%), and Nanog (15.5%) in glioblastoma subsphere samples and the co-expression of CD133 with GFAP (52.5%) and Nestin (44.8%). Representative figure of five samples of glioblastoma.

**Figure 5:**
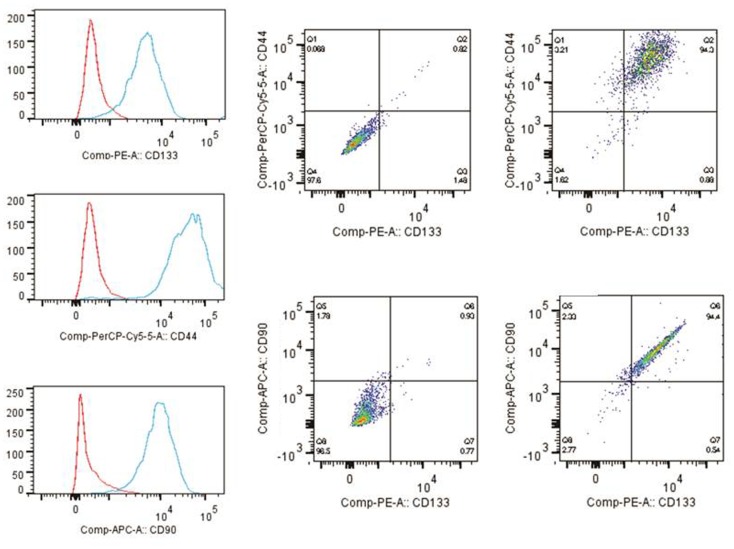
**Immunophenotyping of tumor subspheres by flow cytometry**. The immunophenotypic profile of CD133^+^ cells showed that these cells also expressed high levels of markers CD44 (94.0%) and CD90 (94.4%). Histogram: cells expressing high levels of markers CD44, CD90, and CD133. Dot plots: isotype control gated cells in the first column, and in the second column double staining of CD133/CD44 and CD133/CD90.

**Figure 6:**
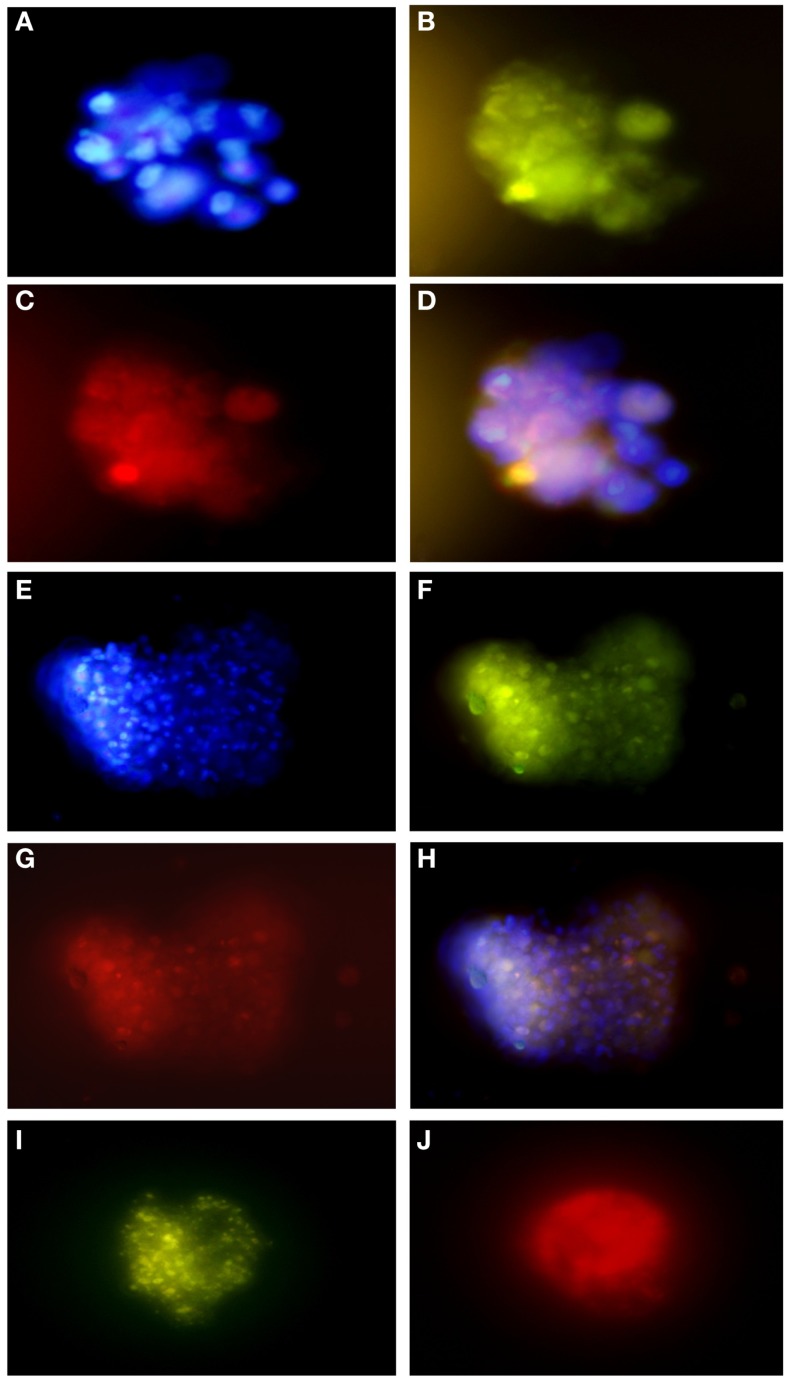
**Immunofluorescence assays of glioblastoma subspheres demonstrate the expression pattern of immunophenotypic markers: (A) DAPI, (B) GFAP, (C) CD133, (D) overlay (A–C); (E) DAPI, (F) GFAP, (G,J) Nestin, (H) overlay, (I) Nanog**. Representative figure of five samples of glioblastoma. **(A–I)** Magnification: 600×.

The tumor subspheres were analyzed by flow cytometry (Figures 4 and 5) and fluorescence microscopy (Figure 6). The flow cytometry analyses showed 92.1% cells expressing GFAP, a tumor glial marker, of which 52.5% double stained for CD133 and 71% cells expressing Nestin, an immature neural stem cell marker, of which 44.8% double stained for CD133. Also, a percentage of these cells also co-expressed Nanog (15.5%) and Sox2 (17.7%), stem cells transcription factors related to pluripotency and self-renewal potential. In addition, the CD133-positive cells also expressed CD44 (94.0%), CD90 (94.4%), and CD34 (34%). CD44 it is described as a homing molecule to lymph node and was previously related with cells metastatic potential, CD90 and CD34 are considered stem cell markers (Figures 4 and 5).

These results corroborate with subsphere immunofluorescence assays showing the same expression pattern of the immunophenotypic markers. We found that most GFAP positive cells (Figures 6B,F) co-expressed CD133 (Figure 6C) and Nestin (Figure 6G), as can be seen by overlap in Figures 6D,H. Double staining also revealed that most Nestin-positive cells (Figure 6J) co-expressed Nanog (Figure 6I).

## Discussion

Despite advances in therapeutics, the molecular and cellular mechanisms that initiate and establish human brain cancer have not been completely elucidated. Recent studies have showed that brain cancer evolved from a specific tumorigenic cell subset with highly self-renew potential called tumor or cancer stem cell (19, 20).

Even though, there isn’t a consensus about the true nature of these cancer stem cells. The cancer stem cell hypothesis is based on the observation that brain tumors are a heterogeneous cell mass that comprise a rare cell population identified by CD133 expression. The CD133-positive cells are able to initiate a new tumor formation and they are responsible for metastasis *in vivo* or neurospheres formation *in vitro* (3).

In order to verify the cancer stem cell hypothesis, it is first necessary to establish preeminent protocols for cells isolation/purification, better cell culture conditions, and procedures for this cell population characterization.

Current protocols in stem cell biology describe a procedure for neurospheres isolation from fresh tumor (3, 17, 19, 21, 22). In addition, our study describes an optimization of CD133-positive cells isolation and the establishment of neurospheres/subspheres from glioblastoma primary culture.

Immunocytochemical and immunofluorescence assays showed CD133-positive cells presence in glioblastoma primary cell culture. Under the electron microscopy, ultrastructural analysis showed the microbead anti-CD133 attached to the selected cells.

The CD133 selected cells were able to further, in cell culture conditions, generate tumor neurospheres/subspheres as a clonogenic set of cells. Our study confirmed the ability of CD133 microbeads to enrich cells with stem-like properties. This finding is important when considering cells heterogeneity in the tumoral mass (23).

CD133-positive cells presence in brain tumor are accepted, even with restriction, as a presence of brain cancer stem cells, which contribute to tumor initiation and recurrence. Furthermore, CD133 expression in brain tumors has been identified as a poor prognosis factor and as critical drivers of tumoral progression, due to their self-renewal capacity and limitless proliferative potential (24–27). A recent study, showed that CD133-positive glioma cells have a molecular profile similar to embryonic stem cells, and CD133-positive glioma cells are more tumorigenic than the CD133 negative population isolated from the same tumor (28). Others, also suggested that CD133 expression could be used as an indicator of brain tumors aggressiveness and dissemination potential, as well as a target for new therapies in glioblastoma (19, 27–29).

There is an overall lack of consensus concerning methodologies for cell sorting and *stemness verification*. Moreover, there is a highly relevant debate about the best method for culturing the human glioblastoma stem cells: some groups proposed the use of adherent monolayer cultures rather than non-adherent cultures, since a homogeneous exposure to growth factors, oxygen and nutrients increases the possibility of obtaining a more homogeneous cell population (15, 30). A study by Motegi et al. suggested that CD133-positive cells can be efficiently isolated from U87MG adherent cultures, which is a malignant glioma cell line (31).

We have modified the CD133 cell population isolation method and neurospheres/subspheres establishment in this study. The glioblastoma primary culture derived neurospheres with 29% more CD133-positive cells when compared to tumor fresh isolated neurospheres. These CD133-positive fractions were able to further generate subspheres. The subspheres derived from glioblastoma primary culture presented a well-defined morphology while the ones derived from the fresh tumor were sparce and less robust. Also, CD133-positive cells derived from glioblastoma primary culture were able to generate 75% more subspheres than the ones derived from the fresh tumor. The negative fraction of CD133 cells was unable to generate subspheres.

Considering these findings, we can suggest that the tumor mass, which has distinct cell types with distinct expressions, comprises adherent and no adherent cells. In our study, we derived neurospheres from glioblastoma primary cultures adherent CD133-positive cells, which the expression was detected by immunocytochemical and immunofluorescence assays. Our findings corroborate another study that attributes to an adherent cell the source of CD133 in glioblastoma (32).

Glioblastoma subpheres analysis revealed that most CD133 isolated cells co-expressed GFAP and Nestin, relating the isolated cells with neural and stem cell origin. GFAP expression reveals glial origin and Nestin characterizes commitment to neural immature lineages (13). Tumor subsphere immunofluorescence assays also revealed that most Nestin-positive cells co-expressed Nanog, leading us to believe that the subspheres also contains undifferentiated neural stem cell (33).

Recently, CD90 another stem cell marker, has been identified as a prognostic marker for high-grade gliomas and CD44 as a metastatic potential marker (34, 35). In addition, CD133, CD90, CD44, and Nestin co-expression may explain the tumorigenic potential of these cells, since Nestin and CD44 are also known as regulators of migration, invasion and tumor growth. Thus, CD133 expression in combination with Nestin, CD44, and CD90, may lead us to a cell profile with highly tumorigenic potential. Consequently, these markers may be useful as a target for molecular therapy in gliomas, including glioblastoma (34–37).

**Figure 7:**
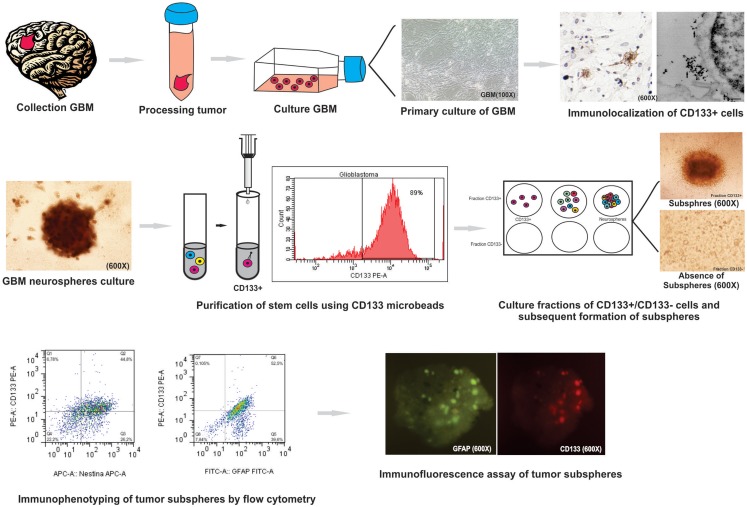
**Summary of protocol parameters for isolation, culture, and immunophenotypic characterization of glioblastoma (GBM) neurospheres derived from tumor primary culture**. This study showed that the method is effective for: (i) the establishment of primary glioblastoma cell culture; (ii) study immunocytochemical and ultrastructural staining of CD133 cells glioblastoma: (iii) culture and isolation of tumor neurospheres derived of primary culture; (iv) purification of the cell population known for initiate the tumor (CD133^+^ cell) by MACS and evaluation by flow cytometry; (v) purification of stem cells using CD133 microbeads by; (vi) formation of subspheres in CD133^+^ cells in neurospheres derived from glioblastoma primary culture; (vii) study of the expression level of GFAP, CD133, Nestin, Nanog, CD34, and Sox2 markers on tumor subspheres by flow cytometry and immunofluorescence assay.

During this study we were able to describe a novel methodology for neurospheres isolation from glioblastoma primary culture through CD133 adherent cells isolation (Figure 7). We were able to generate subspheres from the CD133 isolated cells, which were characterized by its co-expression with GFAP, Nestin, Nanog, CD44, and CD90 establishing the initial and successful method for further studies with these cell populations. These protocols may help further studies about the gliomagenesis process by pinpointing the cell type that is responsible for tumor origin.

## Conflict of Interest Statement

The authors declare that the research was conducted in the absence of any commercial or financial relationships that could be construed as a potential conflict of interest.
